# Epidemiology of acute kidney injury in hospitalized pregnant women in China

**DOI:** 10.1186/s12882-019-1255-8

**Published:** 2019-02-26

**Authors:** Diankun Liu, Wenjuan He, Yanqin Li, Mengqi Xiong, Long Wang, Jingxin Huang, Ling Jia, Shuling Yuan, Sheng Nie

**Affiliations:** The National Clinical Research Center for Kidney Disease, State Key Laboratory of Organ Failure Research, Renal Division, Nanfang Hospital, Southern Medical University, 1838 North Guangzhou Ave, Guangzhou, 510515 China

**Keywords:** Acute kidney injury, Pregnancy, Epidemiology

## Abstract

**Background:**

Epidemiologic data of acute kidney injury (AKI) during pregnancy is lacking in China. This study aims to determine the effect of pregnancy on the risk of AKI among hospitalized women of childbearing age, and to describe the incidence, risk factors and outcomes of AKI in hospitalized pregnant women in China.

**Methods:**

We previously conducted a nationwide, multi-centered cohort of hospitalized patients from 25 hospitals in China during 1/1/2013 to 31/12/2015. Women of childbearing age (14–50 year) who had at least two serum creatinine tests within any 7-day window were selected as analysis set. Patient-level data were obtained from the electronic hospitalization information system and laboratory databases. AKI events were identified according to the creatinine criteria of Kidney Disease Improving Global Outcomes.

**Results:**

Among 110,873 women of childbearing age, pregnant women (*n* = 10,920) had a 51% higher risk of AKI than non-pregnant women (*n* = 99,953). Community acquired and hospital acquired AKI occurred in 3.6% (*n* = 393) and 3.7% (*n* = 402) of the pregnant women, respectively, giving rise to an overall AKI incidence of 7.3%. While, hospital coding would have identified less than 5% of AKI episodes. The top three risk factors of AKI during pregnancy, ranked in order of decreasing population attributable fractions were pregnancy-induced hypertension syndrome (21.1%), acute fatty liver (13.5%), and chronic kidney disease (6.2%).

**Conclusion:**

AKI in pregnancy is associated with increased maternal mortality rate, longer length of stay and higher daily cost. AKI is a common and severe complication during pregnancy in China.

**Electronic supplementary material:**

The online version of this article (10.1186/s12882-019-1255-8) contains supplementary material, which is available to authorized users.

## Background

Acute Kidney Injury (AKI) is a clinical syndrome that primarily present as a rapid decline in kidney function, which is frequent in hospitalized adults and is strongly associated with high mortality and morbidity [1]. Numerous studies have reported the incidence, risk profile and outcomes of AKI in different study populations and clinical settings [2–4]. World Kidney Day and International Women’s Day in 2018 are commemorated on the same day, thus offering an opportunity to highlight the importance of kidney disease in women’s health.

Pregnancy is a unique state for women, where acute or chronic kidney diseases may manifest and impact future generations with respect to kidney health. However, few studies compared the risk of AKI between pregnant women and non-pregnant women. Up to date, there is no population-based study quantified the effect size of pregnancy on the risk of AKI among women of childbearing age.

AKI was regarded as a rare but severe complication during pregnancy, which was associated with increased risk of poor outcomes [5]. Previous studies reported the incidence of pregnancy-related AKI has declined from 1/3000 to 1/20000 in developed countries, which was attributed to the improvement in antenatal care and decline of septic abortion [6]. However, the burden and risk profile of pregnancy-related AKI in developed countries might be different from that in developing world [5, 7, 8]. The reported incidence of AKI during pregnancy in developing countries, such as India, and Pakistan ranged from 0.02 to 11.5% [9–17]. These studies were limited by small sample sizes, highly selected populations, and varying definitions of AKI those have not been validated in pregnancy [9–20]. High-quality epidemiological data on AKI during pregnancy was lacking, particularly in developing world.

We previously conducted a large-scale, multi-centered cohort from 25 centers across China, encompassing a wide range of disease spectrum and severity. Women of childbearing age (14–50 years old) who had at least two serum creatinine (SCr) tests within any 7-day window during hospitalization were selected from the cohort for analysis. The aims of this study were (1) to determine the effect of pregnancy on the risk of AKI among women of childbearing age; (2) to describe the incidence, risk factors and outcomes of AKI in hospitalized pregnant women in China. Evidence from our study may help to increase the awareness of pregnancy-related AKI, and to optimize prevention and intervention of this syndrome in China.

## Methods

### Study design, population and data source

EACH study (Epidemiology of AKI in Chinese Hospitalized patients) was a multi-centered retrospective cohort study conducted in 25 regional medical centers from 15 provinces across China. The study cohort included 3,044,224 patients admitted from January 1, 2013 to December 31, 2015. We selected from the cohort women of childbearing age (14–50 years) who had at least two SCr tests within any 7-day window during their first 30 days of hospitalization as analysis set. We only included SCr tests that used an enzymatic assay and excluded patients with end-stage renal disease (ESRD), receiving maintenance dialysis or renal transplantation. For patients with multiple hospitalizations, we only included the first hospitalization in the analysis set. Pregnancy and its related comorbidities were identified by the diagnostic code. Delivery were identified by the operation procedures.

We obtained patient-level data from the electronic hospitalization databases and laboratory databases in the participating centers. The hospitalization records consisted of patients’ age, sex, date and diagnostic code at admission and discharge, operation procedures and dates, need for intensive care, in-hospital death, and total hospitalization cost. The laboratory data included time of patients’ SCr tests and value of SCr. The Medical Ethics Committee of Nanfang Hospital approved the study protocol and waived patient consent. While the approval number is NFEC-2014-098.

### Identification and classification of AKI

AKI was defined as an increase in SCr by 26.5 μmol/L (0.3 mg/dl) within 48 h or a 50% increase in SCr from the baseline within 7 days according to the Kidney Disease Improving Global Outcomes (KDIGO) criteria [21]. We screened patients’ SCr for the onset of AKI using an algorithm described previously [22]. At any time point t, a baseline SCr was dynamically defined as the mean of SCr levels within 30 days prior to t, and each of the available SCr data within 7 days after t was compared with this baseline. The earliest day when the SCr change met the KDIGO criteria was defined as the date of AKI onset. Patients who met at least one of the following criteria were classified as having community acquired AKI [22]: (a) admitted with AKI according to diagnostic code; (b) having multiple creatinine tests in the clinic, and comparing to the previous measurement, the increase in creatinine on the first day of hospitalization met the KDIGO definition; and (c) SCr on admission ≥1.5 fold of standardized SCr reference value and ≥ 1.5 fold of the minimal SCr level during hospitalization. In these cases, the lowest SCr during hospitalization was used as the baseline creatinine level for patients with community acquired AKI, and the mean SCr during the first 30 days of hospitalization for those without AKI. Cases that met the KDIGO creatinine criteria but not the criteria for the community acquired AKI were identified as having hospital acquired AKI.

### Outcomes and comorbidities

The primary outcome of AKI was in-hospital death. Other outcomes included adverse perinatal outcomes (containing preterm delivery, abortion, dystocia and stillbirth), length of stay in hospital and daily cost of hospitalization. Primary diagnosis and coexisting comorbidities (including chronic kidney disease, convulsion, chronic obstructive pulmonary disease, diabetes mellitus, diarrhea, gastrointestinal bleeding, peptic ulcer disease, heart failure, hematological tumor, hypertension, liver disease, pulmonary infection, myocardial infarction, glomerulonephritis, pregnancy-induced hypertension syndrome, acute fatty liver, sepsis, shock, systemic lupus erythematosus, stroke, thrombotic microangiopathy, trauma, hyperlipidemia)were identified by the diagnostic codes (ICD10-CM codes, see Additional file 1).

### Statistical analysis

Quantitative data was expressed as mean ± SD. Data that do not meet the normal distribution was expressed as median and interquartile range; Categorical data was expressed as frequencies and percentages (n, %). Baseline characteristics of study population were compared using ANOVA test for continuous variables and χ2 tests for categorical variables. The Wilcoxon rank sum test was used for continuous variables that were not normally distributed. Logistic regression model was used to estimate the odds ratio (OR) of risk factors for AKI in hospitalized pregnant women, with adjustment of age, baseline SCr, length of stay in hospital, division, hospital and clinical comorbidities. We calculated the population attributable fractions (PAF) using the formula PAF = f(r-1)/[1 + f(r-1)], where r is the estimated relative risk and f is the proportion of AKI cases that were exposed to the risk factor of interest. We estimated the hazard ratio (HR) of AKI and other possible risk factors for in-hospital death in pregnant women with AKI using Cox proportional hazard model. The value of *P* < 0.05 was considered statistically significant. All the statistical analysis was performed using SPSS version 20.0 for windows (SPSS Inc., Chicago, IL, USA).

## Results

### Baseline characteristics of study population

**Fig. 1:**
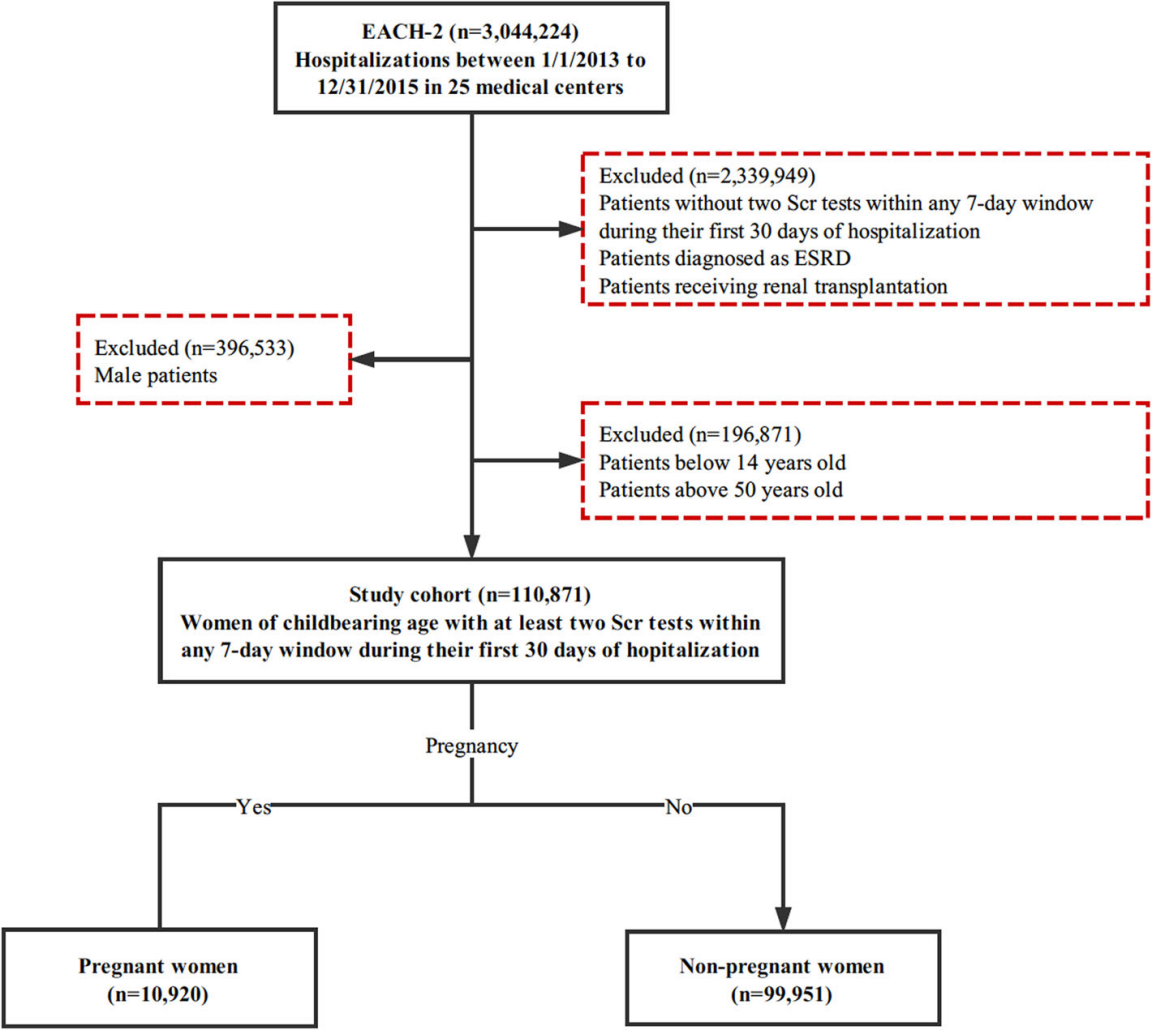
Flowchart of study population selection

Among 110,873 hospitalized women of childbearing age (14–50 years old), a total of 10,920 pregnant women were included as analysis set (Fig. 1). We identified 795 AKI events from the pregnant women (in Additional file 2: Table S1). The characteristics of pregnant women stratified by AKI status were shown in Table 1. Compared with the non-AKI group, AKI group had a higher percentage of patients requiring intensive care and patients with preexisting chronic kidney disease (CKD), hypertension and systemic lupus erythematosus. Women who developed community acquired AKI had a higher baseline SCr compared to those without AKI. Considering the pregnancy-related complications, pregnancy-induced hypertension (PIH) syndrome (including gestational hypertension, preeclampsia, eclampsia) was more prevalent in AKI group (41.0% in community acquired AKI, 23.1% in hospital acquired AKI). The proportion of PIH and acute fatty liver (AFL) in women with community acquired AKI were significantly higher than that in women without AKI and women with hospital acquired AKI.

**Table 1:** Clinical characteristics of pregnant women stratified by AKI status

Variables	Non-AKI *N* = 10,264	CA-AKI *N* = 398	HA-AKI *N* = 411	*P*-value^*^
Age	30.3 (5.6)	29.7 (6.1)	30.2 (5.7)	0.13
< 35	8015 (79.2)	309 (78.6)	320 (79.6)	
≥ 35	2106 (20.8)	82 (21.4)	84 (20.4)	
Baseline SCr	52.9 (43,58)	67 (53,101)	46 (35,65)	< 0.001
Need for ICU	196 (1.9)	61 (15. 5)	24 (6.0)	< 0.001
Dialysis	3 (0.03)	4 (1.0)	2 (0.5)	< 0.001
Preexisting medical conditions				
CKD	255 (2.5)	48 (12.2)	27 (6.7)	< 0.001
Diabetes mellitus	1001 (9.9)	16 (4.1)	45 (11.2)	< 0.001
Hypertension	481 (4.8)	62 (15.8)	34 (8.5)	< 0.001
Systemic lupus erythematosus	137 (1.4)	9 (2.3)	12 (3.0)	0.01
Pregnancy complications				
PIH	1486 (14.7)	161 (41.0)	93 (23.1)	< 0.001
Acute fatty liver	20 (0.2)	33 (8.4)	6 (1.5)	< 0.001
Hyperlipidemia	736 (7.3)	25 (6.4)	32 (8.0)	0.68
TMA	23 (0.2)	5 (1.3)	1 (0.2)	< 0.001
Heart failure	191 (1.9)	21 (5.3)	22 (5.5)	< 0.001

Age is expressed in mean (SD); Baseline SCr is expressed in median (q25, q75), other data are expressed in n (%)

*Non-AKI* without acute kidney injury, *CA-AKI* community-acquired acute kidney injury, *HA-AKI* hospital-acquired acute kidney injury, *SCr* Serum creatinine, *ICU* intensive care unit, *CKD* chronic kidney disease, *PIH* pregnancy induced hypertension syndrome, *TMA* thrombotic microangiopathy

^*^P value are calculated by chi-square test and ANOVA test for categorical data and quantitative data, respectively. Wilcoxon rank sum test is used for continuous variables that were not normally distributed

### Incidence of pregnancy-related AKI

**Fig. 2:**
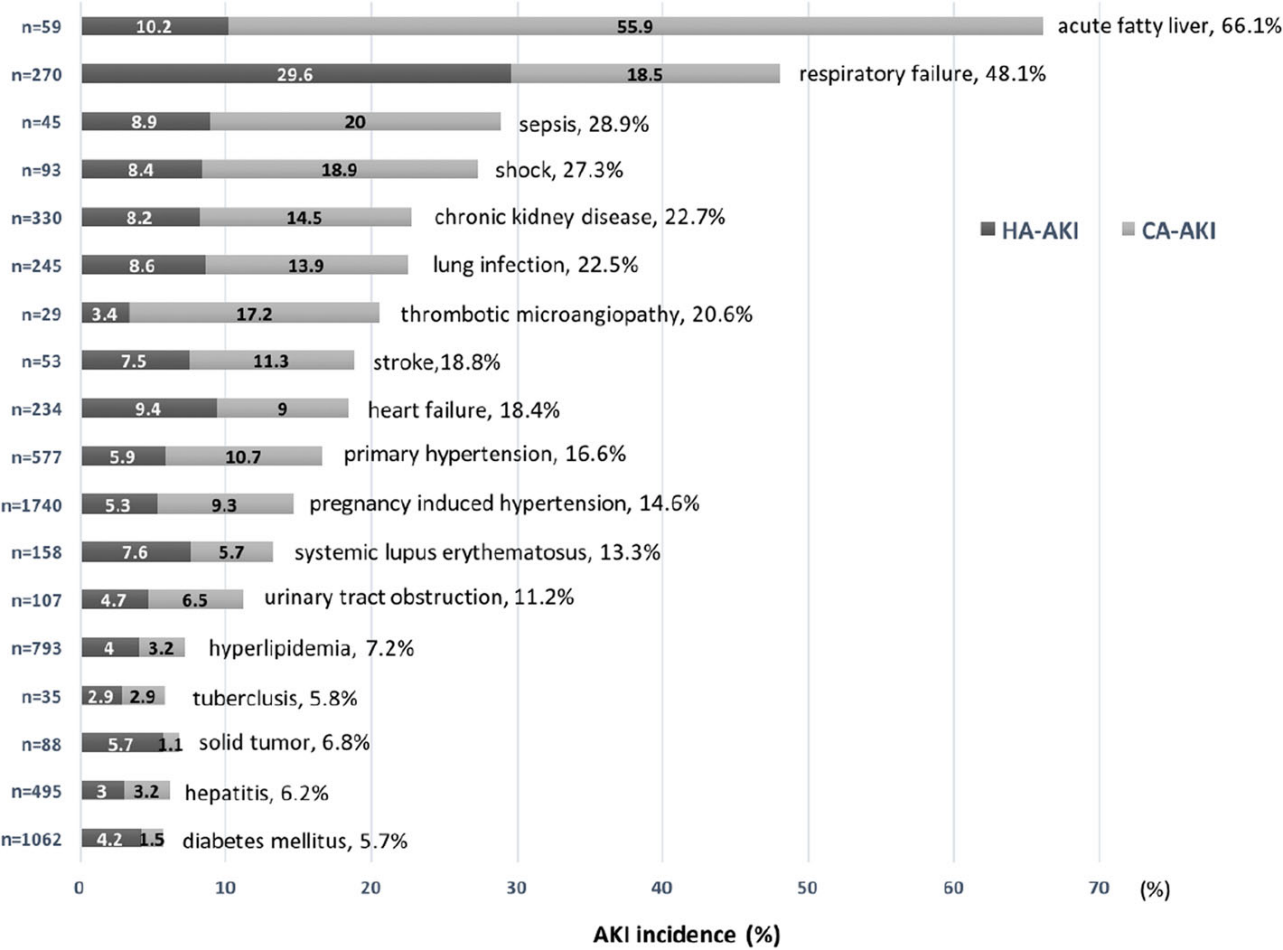
Incidence of AKI in various clinical settings. The number of patients with a clinical setting is indicated by n. HA-AKI, hospital-acquired AKI; CA-AKI, community-acquired AKI

Of 10,920 pregnant women, community acquired AKI and hospital acquired AKI occurred in 3.6% (*n* = 393) and 3.7% (*n* = 402) of the population in the analysis set, respectively, giving rise to an overall incidence of 7.3%. The incidence of community acquired and hospital acquired AKI in various clinical settings was depicted in Fig. 2. The three clinical settings with the highest incidence of community acquired AKI were acute fatty liver (55.9%), sepsis (20.0%) and shock (18.9%). For hospital acquired AKI, the top three clinical settings were respiratory failure (29.6%), acute fatty liver (10.2%), and heart failure (9.4%). Only 4.0% (*n* = 32) of AKI events, which were identified by SCr change, were diagnosed on the discharge record.

### Effect of pregnancy on the risk of AKI among women of childbearing age

Among 99,953 non-pregnant women of childbearing age (14–50), we calculated the incidence of AKI at 6.0%. Compared with the non-pregnant group, pregnant women had a 51% increased risk of AKI (Odds Ratio [OR] 1.51, 95% Confidence Interval [CI] 1.35 to 1.69), after adjusting for age, baseline SCr, length of stay in hospital, division, hospital, need for intensive care, and clinical comorbidities (in Additional file 2: Table S1).

### Risk factors for pregnancy-related AKI

The risk profile of AKI during pregnancy is presented in Table 2. Advanced maternal age was not a significant risk factor for pregnancy-related AKI. We estimated the population attributable fractions (PAF) of the risk factors to assess their contribution to AKI during pregnancy. Overall, the top three risk factors, ranked in order of decreasing PAF, were PIH (21.2%), AFL (13.5%), and CKD (6.2%) for AKI during pregnancy.

**Table 2:** Risk profile of AKI in pregnant women

Variables	Frequency (%)	Odds Ratio (95%CI)*	PAF (%)
Age			
< 35	79.2		
≥ 35	20.8	1.04 (0.87–1.25)	ref
Clinical settings			
CKD	3.0	3.20 (2.38–4.29)	6.19
Diabetes mellitus	9.7	0.79 (0.60–1.04)	−2.07
Heart Failure	2.1	2.46 (1.70–3.55)	2.97
Hypertension	5.3	1.61 (1.24–2.09)	3.13
Lung infection	2.2	2.66 (1.87–3.77)	3.52
SLE	1.4	1.39 (0.84–2.29)	0.54
TMA	0.3	2.67 (0.97–7.33)	0.50
Sepsis	0.4	3.66 (1.74–7.69)	1.05
Shock	0.9	6.19 (3.83–9.99)	4.46
PIH	15.9	2.68 (2.26–3.19)	21.12
Acute fatty liver	0.5	32.18 (18.37–56.35)	13.49
Hyperlipidemia	7.3	1.15 (0 .86–1.55)	1.08

^*^adjusted for age, baseline creatinine, length of stay in hospital, division, hospital and clinical comorbidities

*PAF* population attributable fractions, *OR* odds ratio, *CI* confidence interval, *ICU* intensive care unit, *CKD* chronic kidney disease, *SLE* systemic lupus erythematosus, *PIH* pregnancy induced hypertension syndrome, *TMA* thrombotic microangiopathy

### In-hospital outcomes of pregnant women with AKI

In-hospital outcomes and adverse perinatal outcomes of pregnant women were illustrated in Table 3. The incidence of in-hospital death was 0.1, 1.0, 2.1, and 7.4% in non-AKI, patients with AKI stage 1, 2, and 3, respectively (in Additional file 2: Table S2). The Hazard Ratio (HR) of in-hospital death adjusted for age, baseline SCr, length of stay in hospital, division, hospital, need for intensive care, and clinical comorbidities was 6.8 (95%CI, 2.8 to 16.2) for patients with AKI. Pregnancy-related AKI was associated with longer length of stay in hospital and higher daily cost during hospitalization. The average cost in pregnant women with AKI is 83% higher than that in patients without AKI. The average length of stay was 11 days in AKI patients and 9 days in non-AKI patients. The incidence of adverse perinatal outcomes was 23.9% (*n* = 190) and 22.8% (*n* = 2313) in hospitalized pregnant women with and without AKI, respectively (*P* = 0.485). Compared to patients without AKI, pregnant women with AKI are more likely to have stillbirth (1.0% in AKI group versus 0.2% in non-AKI group, *P* < 0.05), while the risk of preterm-delivery, abortion and dystocia showed no significant difference.

**Table 3:** In hospital outcomes and adverse perinatal outcomes in pregnancy related AKI

Variables	Non-AKI	AKI	*P* value
All pregnant woman (*n* = 10,920)	10,125	795	
In-hospital death	13 (0.1%)	17 (2.1%)	< 0.001
Cost (Ұ)	12,606 (6844, 21,258)	23,146 (11,291, 46,932)	< 0.001
Cost ($)	1877 (1019, 3166)	3447 (1681, 6990)	< 0.001
Length of hospital stay (days)	9 (7, 14)	11 (8, 19)	< 0.001
Perinatal complication^a^	2313 (22.8%)	190 (23.9%)	0.485

^a^abnormal birth included preterm delivery, abortion, dystocia and stillbirth

Cost and length of hospital stay are expressed in median (q25, q75), other data are expressed in N (%); Non-AKI: without acute kidney injury; LOS: Length of stay in hospital

## Discussion

This study represented the extensive epidemiological description of AKI among hospitalized pregnant women in China, encompassing a wide range of disease spectrum and severity. We determined the adverse effect of pregnancy on the risk of AKI among women of childbearing age. We estimated a cumulative incidence of 7.3% for pregnancy-related AKI using the KDIGO creatinine criteria. We calculated the contributions of potential risk factors to AKI during pregnancy. We demonstrated that pregnancy-related AKI was associated with higher risk of in-hospital death and increased resource utilization.

Up to date, there is no consensus on the diagnostic criteria of pregnancy-related AKI among nephrologists and obstetricians. Physiologic changes on hemodynamics result in hyper-filtration of the glomerulus and decrease of SCr during pregnancy, which is most significant in the second trimester [23]. SCr above 70.72 μmol/L is considered abnormal in pregnant women in most of the previous studies [19, 23]. Some studies considered that pregnant women with SCr level above 70.72 μmol/L and no previous history of CKD should be diagnosed as pregnancy-related AKI [9]. Using a fixed upper limit value to define AKI, which did not take into account of the relative change of SCr within a time period, will miss the mild AKI events in patients with low baseline SCr. Furthermore, without the comparison of SCr change, it is hard to distinguish preexisting CKD from AKI, especially when the previous medical history is not available.

The KDIGO criteria, as standardized criteria of AKI, has been widely adopted by professional societies and validated in various study population. However, KDIGO definition has not been broadly applied in pregnant women. In our study cohort, KDIGO-defined AKI was associated with significant increased risk of in-hospital death, longer length of stay in hospital, and higher cost in hospital. Our results suggested that KDIGO creatinine criteria could be used as standardized criteria to define AKI during pregnancy. Using the uniform criteria could make it possible to evaluate and compare the incidence and clinical effect of pregnancy-related AKI across countries and studies.

In our cohort, the cumulative incidence of AKI during pregnancy was 7.3%, which was significantly higher than that among non-pregnant women. After adjustment for confounders, we identified that pregnancy was an independent risk factor of AKI among women of childbearing age and quantified the effect size. AKI was regarded as a rare complication during pregnancy, our result demonstrated that AKI was common and severe which should be paid more attention to during pregnancy.

The previous single-centered studies reported that the incidence of AKI ranged from 0.12–2.51% in pregnant women from China [8, 9, 17]. Notably, most of these studies identified the AKI event by using diagnostic codes, which might under-estimate the incidence of AKI [7, 24]. Our study is the first multi-centered study regarding pregnancy-related AKI in China with a wide coverage of geographic regions and a strict adherence to the KDIGO SCr criteria. Our results provide an extremely important supplement, as well as extension, to previous single-centered studies with respect to AKI during pregnancy and puerperium.

Our previous study revealed that CKD is the leading etiology for AKI in the general population [23]. However, the profile of risk factors for pregnancy-related AKI was unique compared to other populations and has changed overtime. Septic abortion was previously thought as the leading cause of AKI during pregnancy and puerperium in developing countries, such as India and Pakistan [13, 15, 16]. As a result of the improvement of obstetrical care, the risk of AKI due to septic abortion has declined in China. PIH turned into the most common etiology of pregnancy-related AKI due to changes in the lifestyles, higher body mass index, older age of primipara, and increased use of reproductive technology resulting in multiple gestations in contemporary population [8, 9, 22].

AKI during pregnancy is associated with increased mortality, higher cost and longer hospital stays in our study. It is consistent with previous studies from Canada, India and Africa. With the development of obstetrical care, the maternal mortality rate in China has significantly decreased during the last decades. However, *Hildebrand M* [7] and *Prakash J* [25] have both reported that pregnancy-related AKI is associated with adverse perinatal outcomes including preterm-delivery, abortion, dystocia and stillbirth. In our study, AKI in pregnant women is also associated with higher incidence of stillbirth (1.0% vs 0.2%, *P* < 0.05). Although the mechanism behind that is unknown, more attention should be paid on pregnancy-related AKI and its influence on the neonates, as well as long-term kidney outcomes of next generation.

In addition, among 795 pregnancy-related AKI identified by screening the SCr data in our study, only 4.1% of the patients were diagnosed as AKI on discharge records, suggesting that the majority of AKI events were not recognized by clinicians. The low awareness of pregnancy-related AKI in clinical practice may lead to insufficient treatment and monitoring. Given the adverse impact of pregnancy-related AKI on the maternal and neonatal health, there is a clear need for higher awareness, timely diagnosis and proper management of AKI during pregnancy.

The major strength of this study is the large scale of study population encompassing a wide range of disease spectrum and severity. The availability of patient-level data permitted a detailed examination of risk profiles for AKI in pregnant women and statistical adjustment for important confounders. The quantification of risk factors’ contributions provided stronger evidence for preventing AKI at the primary care level.

Our study has several limitations. First, without the clinical information of infants, we could not link the pregnancy-related AKI to the adverse outcomes of neonates. Second, data of urine output is not available in our study, we were not able to validate the effectiveness of KDIGO urine output criteria for defining AKI in pregnant women. The incidence of pregnancy-related AKI will be under-estimated without the use of urine output criteria. Third, most of the hospitalized pregnant women did not repeat the SCr test during hospitalization, making it impossible to identified the AKI events in these patients. Moreover, diagnosis of other comorbidities was from diagnostic code in the electronic database, prospective cohorts are required to further identify and confirm the risk factors for pregnancy-related AKI. Finally, our study cohort was based on the inpatients from tertiary hospitals. The pregnant women included in our study were more severe than normal, which might limit the generalizability of our findings.

## Conclusion

Our study demonstrates that pregnancy is an independent risk factor of AKI among women of childbearing age. The incidence of pregnancy-related AKI is 7.3% among hospitalized women in China. The burden of pregnancy-related AKI was previously under-estimated. PIH is the most common risk factor for developing AKI in pregnancy. Pregnancy-related AKI is associated with increased risk of mortality, higher medical cost, and longer length of hospital stays. Results from this study may help to increase the awareness of pregnancy-related AKI and to improve AKI-related care in China.

## Additional files

Additional file 1: List of Diagnostic Codes. Primary diagnosis and coexisting comorbidities were identified according to the ICD10-CM codes. (DOCX 14 kb)

Additional file 2: **Table S1.** Risk of AKI among pregnant and non-pregnant women. Compared with the non-pregnant group, pregnant women had a 51% increased risk of AKI after adjusting for age, baseline SCr, length of stay in hospital, division, hospital, need for intensive care, and clinical comorbidities. **Table S2.** Incidence of death in different AKI stage. The incidence of in-hospital death was 1.0, 2.1, and 7.4% in patients with AKI stage 1, 2, and 3, respectively. (DOCX 15 kb)

Additional file 3: List of Participating Hospitals. The detailed information on the members of the EACH study group. (DOCX 14 kb)

## Abbreviations

AFL: Acute fatty liver
AKI: Acute kidney injury
ANOVA: Analysis of variance
CI: Confidence interval
CKD: Chronic kidney disease
EACH: Epidemiology of AKI in Chinese Hospitalized patients
ESRD: End-stage renal disease
HR: Hazard ratio
KDIGO: Kidney Disease Improving Global Outcomes
OR: Odds ratio
PAF: Population attributable fractions
PIH: Pregnancy-induced hypertension
SCr: Serum creatinine
