# Supplementary material for: Epidemiology of acute kidney injury in hospitalized pregnant women in China
Source: BMC Nephrol. 2019 Feb 26;20:67. doi: 10.1186/s12882-019-1255-8 (PMC6390352; doi:10.1186/s12882-019-1255-8)
Supplement: Supplementary file 1 — List of Diagnostic Codes. Primary diagnosis and coexisting comorbidities were identified according to the ICD10-CM codes. (DOCX 14 kb) [file 12882_2019_1255_MOESM1_ESM.docx]

**List of Diagnostic Codes**

**Congenital heart disease/ Cardiac surgery:** Q24.9, O99.89, I97.110, I97.120, I97.130, I97.710, I97.790, I97.810, I97.820

**Glomerulonephritis:** N00.2, N00.3, N00.4, N00.5, N00.7, N01.2, N01.4, N01.5, N01.7, N02.2, N02.3, N02.4, N02.5, N02.7, N03.2, N03.3, N03.4, N03.5, N03.7, N04.2, N04.3, N04.4, N04.5, N04.7, N05.2, N05.3, N05.4, N05.5, N05.7, N06.2, N06.3, N06.4, N06.5, N06.7, N07.2, N07.3, N07.4, N07.5, N07.7

**Respiratory failure**: J95.821, J95.822, J96.00, J96.01, J96.02, J96.10, J96.11, J96.12, J96.20, J96.21, J96.22, J96.90, J96.91, J96.92, P28.5

**Shock**: A48.3, O03.31, O03.81, O04.81, O07.31, O08.3, O75.1, R45.7, R57.0, R57.1, R57.8, R57.9, R65.21, T75.01, T78.2, T79.4, T81.1, T81.11, T81.12, T81.19, T88.2, Y63.4, Y84.3

**Heart failure:** I09.81, I11.0, I13.0, I13.2, I50.2, I50.3, I50.4, I50.8, I50.9, I97.13

**Urinary tract obstruction**: N13.0, N13.2, N32.0, N13.5, N13.8, N13.9

**Diabetes**: E08.00, E08.01, E08.1, E08.2, E08.3, E08.4, E08.5, E08.6, E08.8, E08.9, E09.0, E09.1, E09.2, E09.3, E09.4, E09.5, E09.6, E09.8, E09.9, E10.1, E10.2, E10.3, E10.4, E10.5, E10.6, E10.8, E10.9, E11.0, E11.1, E11.2, E11.3

**Diarrhea**: K58.0, K59.1, P78.3, R19.7

**Dehydration**: P74.1, T67.3, E86.0

**Sepsis**: A02.1, A22.7, A26.7, A32.7, A40, A41.0, A41.1, A41.2, A41.3, A41.4, A41.5, A41.8, A41.9, A42.7, A54.86, B37.7, O03.37, O03.87, O04.87, O07.37, O08.82, O85, P36, R65.2

**Respiratory infection**: J06.9, J22, J44.0, J47.0

**Intestinal obstruction:** K50.012, K50.112, K50.812, K50.912, K51.012, K51.212, K51.312, K51.412, K51.512, K51.812, K56.5, K56.60, K56.69, K91.3, P76

**Trauma**: G89.11, G89.21, H05.33, H05.42, H40.3, H61.31, K08.11, K08.41, K08.81, K08.82, M18.3, M87.2, O71.8, O71.9, T79.8, T79.9, Z87.828, Z91.49

**Burn:** T20.00, T20.01, T20.02, T20.03, T20.04, T20.05, T20.06, T20.07, T20.09, T20.1, T20.2, T20.3, T21.0, T21.1, T21.2, T21.3, T22.0

**Anemia**: D46.0, D46.1, D46.2, D46.4, D50, D51, D52, D53, D55, D56, D57.4,

D58, D59, D61,D62, D63, D64

**Malnutrition**: E43, E44, E45, E46, E64.0, O25.1, O25.2, O25.3

**Epilepsy**: G40.00, G40.01, G40.1, G40.2, G40.3, G40.4, G40.80, G40.90, G40.91, G40.B0, G40.B1, Z82.0

**Hypoxic ischemic encephalopathy**: P91.60, P91.61, P91.62

**Urinary tract infection:** N39.0, P39.3

**Preterm**: O42.01, O42.11, O42.91, O60.1, O60.2, P07.3, P59.0

**Hematological malignancy**:C90, C91, C92, C93, C94, C95, C81, C82, C83, C84, C85, C86
