# Supplementary material for: Epidemiology of acute kidney injury in hospitalized pregnant women in China
Source: BMC Nephrol. 2019 Feb 26;20:67. doi: 10.1186/s12882-019-1255-8 (PMC6390352; doi:10.1186/s12882-019-1255-8)
Supplement: Supplementary file 2 — Table S1. Risk of AKI among pregnant and non-pregnant women. Compared with the non-pregnant group, pregnant women had a 51% increased risk of AKI after adjusting for age, baseline SCr, length of stay in hospital, division, hospital, need for intensive care, and clinical comorbidities. Table S2. Incidence of death in different AKI stage. The incidence of in-hospital death was 1.0, 2.1, and 7.4% in patients with AKI stage 1, 2, and 3, respectively. (DOCX 15 kb) [file 12882_2019_1255_MOESM2_ESM.docx]

**Additional file 2**

| **Table S1. Risk of AKI among pregnant and non-pregnant women** | | | | |
| --- | --- | --- | --- | --- |
| **variable** | **N** | **AKI events** | **Incidence** | **Odds Ratio* (95%CI)** |
| Pregnant | 10920 | 795 | 7.3% | 1.51(1.35-1.69) |
| Non-pregnant | 99953 | 6081 | 6.0% | ref |
| * adjusted for age, baseline creatinine, length of stay in hospital, division, hospital and clinical comorbidities.  AKI: acute kidney injury; CI: confidence interval | | | | |

| **Table S2. Incidence of death in different AKI stage** | | | |
| --- | --- | --- | --- |
| **AKI-stage** | **N** | **Death** | **Incidence** |
| Stage-1 | 498 | 5 | 1.0% |
| Stage-2 | 189 | 4 | 2.1% |
| Stage-3 | 108 | 8 | 7.4% |
| AKI: acute kidney injury. | | | |
