# Supplementary material for: Epidemiology of acute kidney injury in hospitalized pregnant women in China
Source: BMC Nephrol. 2019 Feb 26;20:67. doi: 10.1186/s12882-019-1255-8 (PMC6390352; doi:10.1186/s12882-019-1255-8)
Supplement: Supplementary file 3 — List of Participating Hospitals. The detailed information on the members of the EACH study group. (DOCX 14 kb) [file 12882_2019_1255_MOESM3_ESM.docx]

**List of Participating Hospitals**

1. Nanfang Hospital, Southern Medical University, Guangzhou, China;
2. The First Affiliated Hospital of Zhengzhou University, Zhengzhou, China;
3. West China Second University Hospital, Sichuan University, Chengdu, China;
4. Sichuan Provincial People’s Hospital, University of Electronic Science and Technology

 of China, Chengdu, China;

1. Guangdong General Hospital, Guangdong Academy of Medical Sciences, Guangzhou, China;
2. Children’s Hospital of Chongqing Medical University, Chongqing, China;
3. Guizhou Provincial People’s Hospital, Guizhou University, Guiyang, China;
4. The Second Affiliated Hospital, Zhejiang University, Hangzhou, China;
5. Guilin Medical University Affiliated Hospital, Guilin, China;
6. Tongji Hospital Affiliated to Tongji Medical College, Huazhong University of Science and Technology, Wuhan, China;
7. The First Affiliated Hospital, Zhejiang University, Hangzhou, China;
8. The First Affiliated Hospital of Shenzhen University, Shenzhen University, Shenzhen, China;
9. The Second Affiliated Hospital of Dalian Medical University, Dalian, China;
10. Huashan Hospital, Fudan University, Shanghai, China;
11. Zhong Da Hospital, Nanjing, China;
12. Sun Yat-sen Memorial Hospital, Sun Yat-sen University, Guangzhou, China;
13. Children's Hospital of Nanjing Medical University, Nanjing, China;
14. The Children Hospital of Zhejiang University, Hangzhou, China;
15. Anhui Provincial Children's Hospital, Hefei, China;
16. Guangzhou Women and Children's Medical Center, Guangzhou Medical University, Guangzhou, China;
17. Children's Hospital of Fudan University, Shanghai, China;
18. Chengdu Women and Children’s Central Hospital, Chengdu, China;
19. Shanghai Children's Medical Center, Shanghai Jiaotong University, Shanghai, China;
20. Jinan Children's Hospital, Jinan, China;
21. Lanzhou University Second Hospital, Lanzhou, China.
